# Orbital and Pulmonary Actinomycosis: The First Case Report and Literature Review

**DOI:** 10.1155/2018/4759807

**Published:** 2018-07-26

**Authors:** Paruspak Payoong, Natcha Saetiew, Opass Putcharoen, Chusana Suankratay

**Affiliations:** Division of Infectious Diseases, Department of Medicine, Faculty of Medicine, Chulalongkorn University, Bangkok, Thailand

## Abstract

Orbital actinomycosis is a very rare clinical manifestation of orbital infection caused by *Actinomyces* species, anaerobic Gram-positive filamentous bacteria. We report herein a case of a 58-year-old man who presented with chronic progressive course of total ophthalmoparesis in association with productive cough, leading to the diagnosis after extensive investigation. In addition, all reported cases of orbital actinomycosis in the literature are reviewed.

## 1. Introduction

*Actinomyces* species are anaerobic Gram-positive filamentous bacteria and ubiquitous in soil and microbiota of both animals and humans. The infection caused by *Actinomyces* species, actinomycosis, is usually indolent and slowly progressive [1]. There are wide clinical manifestations of actinomycosis depending on the infection site including orocervicofacial, thoracic, abdominal, and pelvic diseases [1]. To date, there have been handful cases of orbital actinomycosis. The source of infection is usually following the head injury or from the infection of adjacent paraorbital organs including paranasal sinus, nose, cavernous sinus, and brain. We report herein a case of a 58-year-old man who presented with chronic progressive course of total ophthalmoparesis in association with productive cough, leading to the diagnosis after extensive investigation.

## 2. Case Report

A 58-year-old Thai previously healthy male farmer, living in Roi Et Province (Northeast Thailand), was referred to our hospital, King Chulalongkorn Memorial Hospital, due to problems with total ophthalmoparesis and productive cough for 2 months. Two months prior to admission (PTA), he developed productive cough and low-grade fever with no response to many courses of antibiotic treatment. One month PTA, he noted binocular horizontal diplopia of the right eye, before progression to visual loss and ptosis despite antibiotic and steroid treatment. His past medical history was unremarkable except heavy alcoholic drinking.

The right eye examination revealed eyelid swelling, proptosis, marked chemosis, and complete ptosis; light perception of visual acuity; absent afferent pupillary reflex; and optic disk swelling and retinal hemorrhage. Neurological examination revealed right eye total ophthalmoparesis and decreased pinprick sensation of right cranial VI nerve area. Multiple dental caries were noted. Pulmonary examination revealed fine crackles at both the lungs. Other examination was unremarkable.

**Figure 1:**
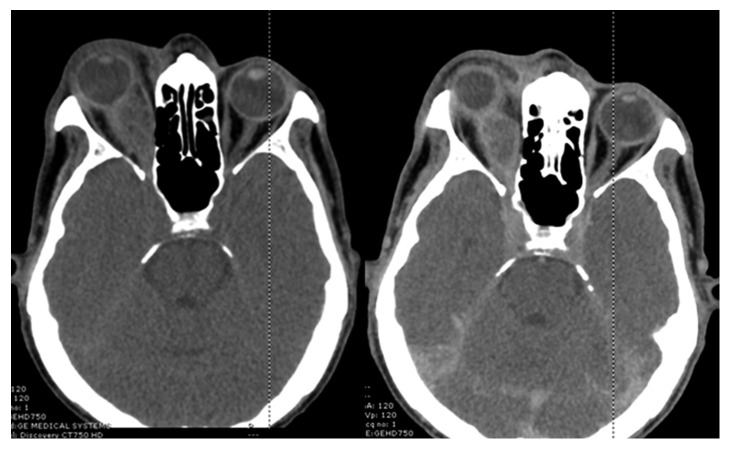
Computed tomography showing rim-enhancing hypodense lesion at the right orbital apex abutting to the optic nerve sheath complex and right medial rectus muscle.

Complete blood count showed hemoglobin of 9.3 g/dL, white blood cells of 25,540 cells/mm^3^ (85.1% neutrophil, 9.8% lymphocyte, and 4.8% monocyte), and platelets of 697,000 cells/mm^3^. Blood chemistry was normal. Anti-HIV test was negative. Chest X-ray revealed bilateral diffuse reticulonodular and alveolar infiltrations. Orbital computed tomography revealed a 1.5 × 2.9 × 1.5 cm rim-enhancing hypodense lesion at the right orbital apex abutting to the optic nerve sheath complex and right medial rectus muscle (Figure 1).

**Figure 2:**
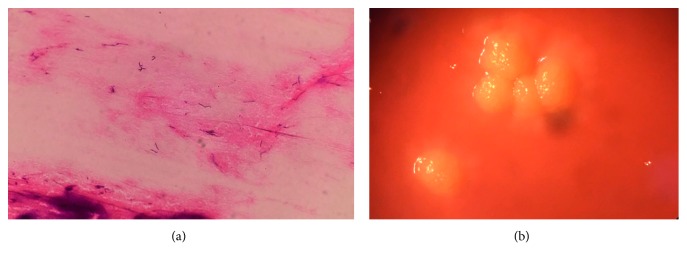
(a) The pus Gram stain exhibiting Gram-positive filamentous bacilli. (b) Molar tooth-like colonies of *Actinomyces israelii*.

A diagnosis of orbital and pulmonary infections was made, and emergent anterior orbitotomy was performed and revealed purulent discharge from the orbit. The pus and sputum Gram stain exhibited many Gram-positive filamentous bacilli, but non-acid-fast, on both standard and modified acid-fast bacilli stains. Hence, *Actinomyces* species was suspected, and *Nocardia* species could be excluded due to negative results on modified AFB stain. The pus cultures finally grew *Actinomyces israelii* (Figures 2(a) and 2(b)). Further bacterial identification by base sequencing of 16S ribosomal DNA was *A. israelii* with 99% identity (NCBI BLAST Search).

He was given penicillin G sodium of 24 million units per day for 6 weeks and amoxicillin of 2 g per day for another 12 months. After 10 days of treatment, there was a much improvement of lung infiltrates on his chest X-ray. The patient was doing well when last seen 1 year after discharge with mild residual impaired ophthalmoparesis and ptosis but still moderate visual impairment.

## 3. Discussion

To our knowledge, our case is the first report of both orbital and pulmonary actinomycosis. All previously reported cases were only orbital actinomycosis.

A portal of entry to the orbit in our case is probably from the hematogenous spread from the lungs, even though only unilateral orbit is involved. Due to sequence of respiratory and eye symptoms, the imaging reveals that there is no infection of paraorbital organs and presumed aspiration pneumonia from heavy alcohol drinking. In contrast, orbital actinomycosis in all previously reported cases was caused by infection in paraorbital organs or following the injury.

A literature review of all 10 cases (including our case) of orbital actinomycosis is shown in Table 1. The median age is 45 (IQR 37, 56) years with the male : female ratio of 2 : 1. Europe and Asia are the most common continents (4, 40% each), followed by North America and South America (1, 10% each). Teeth extraction history and chronic steroid use are the most common predisposing factors (4, 40% each), followed by sinusitis (2, 20%) and trauma (1, 10%). The median duration of illness is 21 (IQR 11, 51) days. Ophthalmoparesis and ptosis are the most common presentations (7, 70% each), followed by proptosis (4, 40%) and visual loss (3, 30%). Six (60%) patients are afebrile. On imaging, there are 5 and 3 extraconal and intraconal lesions (1 infraorbital nerve and 1 unknown data). Of 5 extraconal lesions, there are 3 and 2 intradural and extradural lesions. Of 3 intraconal lesions, there are 2 and 1 central and lateral lesions. Of 10 patients (no data in 1 patient), 8 (80%) undergo surgical intervention. Penicillin is the most common prescribed antibiotic (6, 60%), followed by tetracycline and gentamicin (1, 10% each). There is 12% mortality rate. Full recovery and partial recovery are observed in 2 (20%) and 6 (60%) patients (no data in 2 patients).

## 4. Conclusions

To our knowledge, our case is the first report of both orbital and pulmonary actinomycosis, likely from hematogenous spread from the lungs.

## Figures and Tables

**Table 1:** A summary of all case reports of orbital actinomycosis.

Number	References	Age/sex	Country	Published year	Underlying disease	Presentations, duration (week)	Infection site	Treatment		Outcome
								Surgery	Medication	
1	[2]	42/F	England	1992	Right macular scar	Total ophthalmoparesis, 3	IC (C)	Yes	Metronidazole and high-dose intramuscular penicillin V with probenecid	Improvement after 3 months of follow-up
2	[3]	45/M	India	2001	No	Proptosis and ptosis, 2	EC (ID)	Yes	Crystalline penicillin for 14 days followed by oral tetracycline for 4 weeks	Improvement after 1 month of follow-up
3	[4]	53/F	Italy	2006	Tolosa–Hunt syndrome on methylprednisolone and carcinomas of the kidney and breast	Total ophthalmoparesis, 1	IC (L)	Yes	Benzylpenicillin potassium and benzylpenicillin sodium intravenous for 42 days then amoxicillin oral	Improvement after 1 month of follow-up
4	[5]	32/F	England	1896	No	Total ophthalmoparesis progress to alteration of consciousness, 6	EC (ID)	Yes	Iodide of potassium	Death
5	[6]	54/M	The United States	1932	No	Progressive right temporal and jaw pain, 12	EC (ED)	Yes	NA	Death after 4 months of symptom
6	[7]	20/M	India	2010	History of deep scalp laceration for 1 year	Right eyelid and eyebrow swelling and pain, 2	EC (ED)	Yes	IV penicillin G for 2 weeks then oral amoxicillin for 6 months	Full recovery at 4 months of follow-up
7	[8]	NA	India	2004	NA	Orbital abscess	Orbital abscess	NA	NA	NA
8	[9]	43/M	Italy	2004	No	Total ophthalmoparesis, 1	EC (ID)	No	IV penicillin G then oral amoxicillin	Full recovery at 3 months of follow-up
9	[10]	71/M	Spain	1990	No	Progressive swelling in the subcutaneous tissue overlying the right maxilla, 4	Infraorbital nerve mass	Yes	NA	Improvement
10	Our case	58/M	Thai	2017	No	Total ophthalmoparesis and productive cough, 8	IC (C)	Yes	Penicillin IV for 6 weeks followed by amoxicillin oral 12 months	Improvement on 12 months of follow-up

M: male, F: female, EC: extraconal, IC: intraconal, ED: extradural, ID: intradural, C: central, L: lateral, and IV: intravenous.
